# Interactive Art Design and Color Perception Based on the Sparse Network and the VR System

**DOI:** 10.1155/2022/8348632

**Published:** 2022-09-28

**Authors:** Lie Wang, Jie Luo, Guanlin Luo

**Affiliations:** ^1^School of Fine Arts and Design Department of Product Design, Guangzhou University, Guangzhou 510006, Guangdong, China; ^2^Guangzhou University, Guangzhou 510006, Guangdong, China

## Abstract

At present, with the gradual development of science and technology, people's life has also produced a lot of changes, the traditional communication technology has been gradually changed by the new computer technology, people's life has become more intelligent. However, many current artificial intelligence technologies rely on the promotion of network data. In the mobile terminal, especially in the poor state of some data network environment, many users' intelligence needs cannot be met. From the perspective of user interaction experience, this paper analyzes and investigates the interactive standby link in detail and systematically based on the perspective of context awareness and carries out a battle summary with the scientific, systematic, reasonable, and executable design methods suitable for an interactive standby state and puts forward the recommended items of matrix decomposition. The static information is embedded in the model. The status of information is imported as a dimension different from the previous matrix factorization model, and the accumulation of interaction between user's status conditions and project factors is considered, as well as the sensitivity difference between user and information project status. In order to obtain the global situation of user balance, the project prediction deviation caused by the vector and sensitivity to various conditions is needed. Finally, the training model gets the final prediction score value and puts forward the mobile system user interaction experience art design strategy based on context awareness, which provides a certain idea to meet the needs of mobile system users.

## 1. Introduction

The development of science and technology make people's life go through a very breakthrough change, such as traditional need people carried out through face-to-face social and shopping activities, can now be through the line, people can purchase all kinds of goods and delivery, can also through the network to communicate with people thousands of miles away. With regard to audio and video entertainment, people rely on the replication system to easily obtain their favorite audio and video works from the huge amount of film and music resources. In the aspect of office learning, users can also easily obtain the massive online open-source literature and books they need; in terms of current affairs consultation, today's people really realize the realm of “the scholar does not go out to know the world's affairs,” and everyday users can easily get the political news they are interested in. The abovementioned information technology does not have a profound impact on people's daily life. However, in the highly developed 5 g era, existing data networks cannot realistically meet the rising demands of people, so people are proposing more ideas about life under artificial intelligence. Users are eager for the recommendation system to intelligently identify the dynamic needs of users and the differentiated needs of users in different situations. However, the traditional recommendation methods cannot meet the expectations of intelligent recommendation accuracy.

Here, the influence and function between products and users is bidirectional. First, the use process of these products will also affect the user's use process. The user experience of “interactive standby” is “virtual.” For the psychological and emotional phenomenon, it needs to interact with the needs of related business, functional design, system logic, and cognitive analysis. Is this interactive experience clear, easy to understand, easy to control, easy to get close to, and standard high-speed interactive experience? Interaction design should not only accept the waiting time but also pay attention to the user's feeling, service price, and users' enjoyment of various link services. These factors are indispensable. In the process of standby experience, it is necessary to investigate and learn the main reasons that affect the standby customer experience, to improve and upgrade the products and services, so as to obtain more users.

## 2. Related Work

Facing the urgent practical needs, more and more scholars are committed to the improvement of the context aware recommendation method. Literature recognizes that in practical application of the recommendation system, the user's situation can have a direct impact on the user's interest preference [1]. Therefore, literature directly adopts the way of context information prefiltering or context postfiltering to reflect the impact of static situation on the recommendation results [2]. In reference, a relevance rule recommendation model based on context prefiltering idea is proposed, and the temporal context factor is integrated to realize the relevance rule recommendation algorithm based on temporal context segmentation [3]. The recommendation model proposed in reference adopts the idea of postfiltering according to the situation, and the recommendation accuracy will be improved [4]. However, more scholars are committed to recommending model-based static state information. In reference, considering that the user's interest and the popularity of the project may decrease with time, the time influence factor is added to the deviation item of the model, and a proposal of timesvd++ is proposed [5]. In reference, tensor decomposition is used to integrate context aware implicit feedback information and user's nearest neighbor information in high-dimensional space [6]. However, this method has high computational complexity and is difficult to apply. Reference thinks that the model of the tensor decomposition method is too complex and only effective for variable classification [7]. A context-based recommendation method, factor decomposition machine, is proposed, which can achieve rapid progress. In reference, a matrix decomposition model is proposed to fuse the state information from the bias point of view [8]. For dynamic status information, i.e., comment information, a HFT model was proposed in reference to extract hidden features of the user's comment set or the item comment set by using topic feature extraction technology and merge with hidden content [9]. To improve the accuracy of recommendation, based on the shortcomings of the HFT model, reference has made corresponding improvements [10]. The model designed by literature can capture the content of user comments, analyze the characteristics of data, classify topics and improve prediction accuracy by feature classification [11]. Reference based on collaborative filtering and topic feature modeling is to be recommended [12]. Nowadays, with the popularity of deep learning, it has become a research trend to adopt the corresponding deep learning technology to learn the data characteristics of dynamic situation information. Literature proposed convMF, which uses CNN to process comment text, capture local context information and combine with PMF [13]. In reference, the deepCNN model is proposed [14]. CNN is used to learn the text features of the user comment set and the item comment set, and the score is predicted through the sharing layer. The literature constructs the feature preference matrix of relevant users through deep learning, analyzes the user behavior and predicts the user through the score judgment of the shared layer.

Among the experts and scholars at home and abroad, some people mainly study “standby users” from the perspective of psychology. Combined with empirical research, they explore ways to reduce users' dissatisfaction in standby mode. Reference proposed eight principles of customer standby psychology which are widely recognized and adopted [15]. Literature forms an objective living habit from the perspective of cognition, which is called “queuing psychology” [16]. Literature suggests that “standby psychology is more important than actual standby time” [17]. This reflects that the user's psychological standby time has a greater impact on the user's standby experience than the actual standby time. It feels like time goes by faster than anything. According to the data, people's expectation of waiting time in line is about 36% too low. Literature pointed out that if consumers cannot observe the service process and determine the standby time, the pressure caused by standby will increase monotonously. This kind of psychological pressure can be alleviated by providing consumers with information related to waiting time. According to literature, with the increase of standby time, customer satisfaction after standby decreases. Customers can wait 21 hours for evaluation and provide relevant information. The empirical research in reference also proves that standby may have adverse effects on the evaluation of overall customer service quality.

## 3. Matrix Decomposition Context Awareness Method Based on Deep Learning

### 3.1. Context Aware Recommendation Technology

Accurate definition of context information: context information is context information, which refers to all types of information such as entities, states, emotions, and attributes involved in the interaction between users and projects. The classification and definition of situational data: representative context and interactive context. In this paper, we redefine it as static data and dynamic data. The context aware recommendation system is to add these additional context dimensions to the recommendation system. The condition recognition recommendation system adds these additional situation dimensions to the recommendation system. Condition recognition technology can be realized by three methods: prefiltering, postfiltering, and modeling. The analysis of the three technologies shows that the prefiltering and postfiltering of the situation can only be extended in the static situation data, and the condition maintenance cannot be fully and effectively involved in score prediction. These only serve as scene filtering, so they cannot achieve an excellent performance. The last type of condition modeling idea can effectively utilize the function of condition maintenance in rating prediction and can be developed with two types of condition information data. Therefore, the two models proposed in this paper are based on the idea of condition modeling.

### 3.2. Common Evaluation Indexes of the Recommended Model

In order to prove whether the proposed recommendation model has a good performance and can effectively complete data extraction and analysis, calculation is required. First, the square root error is calculated, as shown in the following formula:

(1) $$\begin{array}{c} RMSE=\sqrt{\frac{\overset{m}{\underset{i=1}{\sum }}{\left(\tilde{R}i-Ri\right)}^{2}}{m}}\text{.} \end{array}$$

Definition of hit rate: the hit rate in recommendation is used to evaluate the recommendation accuracy in the recommendation list provided by the recommendation system, specifically the ratio between the number of recommended hits belonging to the test set of all users and the number of the whole test set. The formula is as follows:

(2) $$\begin{array}{c} HR@K=\frac{NumberofHits@K}{\left|GT\right|}\text{.} \end{array}$$

In the normalized cumulative loss gain (NDCG), it is usually used to measure the consistency between the recommended list and the actual Rankine force. In this subsection, the components are described. First, *CG* is the cumulative gain. In other words, the relevant scores of each recommendation result are accumulated as the overall score of the recommendation list. Assuming the top *k* of the recommended list, its *CG* formula is as follows:

(3) $$\begin{array}{c} CG_{k}=\overset{k}{\underset{i=1}{\sum }}rel_{i}\text{.} \end{array}$$

However, the order of the recommendation list is not considered in the cumulative gain *cG*. Here, the recommendation list is sorted based on the score level, and there is DcG (disrupted cumulative gain), the formula is as follows:

(4) $$\begin{array}{c} DCG_{k}=\overset{k}{\underset{i=1}{\sum }}\frac{2^{reli}-1}{\log_{2}\;\left(i+1\right)}\text{.} \end{array}$$

The recommendation system needs to integrate the recommendation list results of all users. Here, the DCG can be normalized to get NDCG. It is formulated as

(5) $$\begin{array}{c} NDCG_{k}=\frac{DCG_{k}}{IDCG_{k}}\text{.} \end{array}$$

### 3.3. Construction of a Matrix Factorization Recommendation Model

This article proposes user, project, and context interaction calculation, which refers to the mutual influence of some hidden conditions between users and items; the mutual influence of some hidden conditions between users and items and each context dimension, which combines users/items with each context. The implicit interactions of all the conditions of the dimension are added together to obtain the interaction relationship between the user/item and the context dimension. In the case of introducing the interactive calculation of the condition dimension, the model can be divided into three steps. Dialogue between user and project. The interaction between the user and the context and the interaction between the project context. The calculation of the interaction between the user and the project can be solved using the traditional matrix factorization model, as shown in the following formula:

(6) $$\begin{array}{c} {\hat{r}}_{uicl...ck}=\mu +b_{u}+b_{i}+p_{u}^{T}q_{i}+\overset{k}{\underset{L=1}{\sum }}p_{u}^{T}c_{L}+\overset{k}{\underset{L=1}{\sum }}q_{i}^{T}c_{L}\text{.} \end{array}$$

As shown, a matrix decomposition model for calculating the interaction of the situation dimension is introduced. Add user context factor vector and engineering context factor vector. The models are as follows:

(7) $$\begin{array}{c} {\hat{r}}_{uicl...ck}=\mu +b_{u}+b_{i}+\alpha c_{u}+\beta c_{i}p_{u}^{T}q_{i}+\overset{k}{\underset{L=1}{\sum }}p_{u}^{T}c_{L}+\overset{k}{\underset{L=1}{\sum }}q_{i}^{T}c_{L}\text{.} \end{array}$$

Project deviation refers to the change of project scoring benchmarks affected by time and context information, that is, the change in project popularity over time, which usually follows Newton's law of cooling. The calculation is as follows:

(8) $$\begin{array}{c} b_{i\left(t\right)}=b_{i}+b_{i}e^{-\delta t}\text{.} \end{array}$$

User preference items refer to the changes in user rating benchmarks under the influence of time and context information, that is, changes in user interests and preferences over time. The calculation formula is as follows:

(9) $$\begin{array}{c} b_{u}\left(t\right)=b_{u}+\alpha_{u}\cdot dev_{u}\left(t\right)\text{.} \end{array}$$

The calculation of time deviation is as follows:

(10) $$\begin{array}{c} dev_{u}\left(t\right)=sign\left(t-t_{u}\right)\cdot {\left|t-t_{u}\right|}^{\beta u}\text{.} \end{array}$$

The improved model with time factor bias is as follows:

(11) $$\begin{array}{c} {\hat{r}}_{uicl...ck}=\mu +b_{u\left(t\right)}+b_{i\left(t\right)}+\alpha c_{u}+\beta c_{i}p_{u}^{T}q_{i}+\overset{k}{\underset{L=1}{\sum }}p_{u}^{T}c_{L}+\overset{k}{\underset{L=1}{\sum }}q_{i}^{T}c_{L}\text{.} \end{array}$$

In order to obtain the optimal solution of the model, the predicted value can be obtained. We set the loss function as follows:

(12) $$\begin{array}{c} \begin{array}{c} {\hat{r}}_{uicl...ck}=\mu +b_{u\left(t\right)}+b_{i\left(t\right)}+\alpha c_{u}+\beta c_{i}p_{u}^{T}q_{i}+\overset{k}{\underset{L=1}{\sum }}p_{u}^{T}c_{L}+\overset{k}{\underset{L=1}{\sum }}q_{i}^{T}c_{L}\\ C=\sum_{uil}\left(r_{uicl...ck}-\right.{\left.{\hat{r}}_{uicl...ck}\right)}^{2}={\underset{uil}{\sum }\left({r_{ui}-\mu -b}_{u\left(t\right)}-b_{i\left(t\right)}-\alpha c_{u}-\beta c_{i}-p_{u}^{T}q_{i}-p_{u}^{T}c1-q_{i}^{T}c2\right)}^{2} \end{array}\text{.} \end{array}$$

Two open datasets are used in the experiment. In the first article, we use the LDOS comoda dataset. The data is obtained from the user's evaluation of the movie after watching the movie, including the relevant status information. In the second paper, the algorithm is evaluated and validated using the movie lens movie music data set published by the University of Minnesota. In the experiment, as shown in the following formula:

(13) $$\begin{array}{c} RMSE=\sqrt{\frac{1}{\left|T\right|}\overset{m}{\underset{i=1}{\sum }}{\left(\tilde{R}i-Ri\right)}^{2}.} \end{array}$$

The results show that RMMSE (root mean square error) is used as the evaluation benchmark of the model. This paper contains several variables. And the use of information on the use of the Ka model to achieve the use of information.  Table 1 shows the information gain values of each scene recognition in the LDOS comoda dataset.

Compared with the traditional matrix decomposition model SVD++, LFM, the prediction accuracy of the proposed model has been significantly improved in the same data set environment. The performance improvement of the proposed model is 3.1% and 4.6% for the two comparison models in the 1ok dataset, and 11.9% and 13.2% in the LDOS dataset. It shows that the model effectively combines the situational information beyond the score and has a positive effect on the prediction score. In the experimental data set with more static context dimensions, the effect of this model is improved more than the existing models, which shows that the model in this paper can make full use of situational information. Meanwhile, considering the deviation caused by different users and projects' sensitivity to situational variables, we should try our best to avoid invalid context information on the final recommendation result under the premise of maximizing the use of situational information, in order to get a more accurate and an efficient recommendation model.

### 3.4. Construction and Result Analysis of the Multi-Source Feature Hybrid Recommendation Model

**Figure 1:**
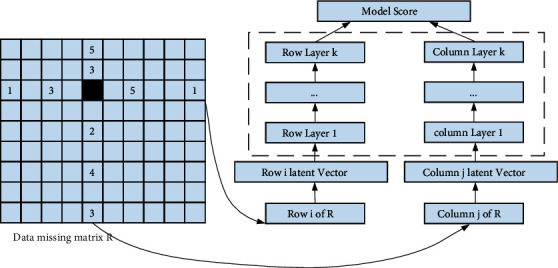
Module structure of deep feature extraction of the scoring matrix.

In the existing recommendation algorithms based on matrix decomposition, most of them decompose the rating matrix into the inner product of two low rank matrices to predict the user rating. This kind of a shallow model can only obtain the linear characteristics of users and items in the rating matrix and essentially reflects the linear relationship between users and items. In the complex and sparse rating data of large-scale recommendation system, the effect of traditional shallow linear model is not ideal. In this paper, deep learning technology is applied to the matrix decomposition module. The missing fractional rows and columns are used as potential feature inputs, and the deep nonlinear interaction between users and projects based on the fractional matrix is obtained by nonlinear transformation of the multilayer insight accelerator. In this paper, the depth feature extraction module of the fractional matrix includes input layer, embedding layer, multilayer, insight layer, and output layer. Insight into the structure of the following layers of modules is introduced. The module structure is shown in Figure 1.

Sparse scoring matrix has a high dimension, and its missing values are often not directly used as input data for deep network learning. In order to get the input vector of appropriate dimension, it is necessary to project the original row and column vector of the missing value to get its low dimensional expression. The embedding layer usually acts as the first layer of the model to reduce the dimension of high-dimensional redundant data. For the highly sparse MXN original score matrix *R*, its missing value *r* corresponding to row and column vectors are *RI* and *RJ*, respectively, which are projected to *k*-dimensional variables through the following nonlinear transformation, such as the following formula:

(14) $$\begin{array}{c} x_{u}=\delta \left(R_{i}*W_{u}\right)\text{,}\\ x_{v}=\delta \left(R_{v}*W_{j}\right)\text{.} \end{array}$$

The learning mechanism of the multilayer perceptron layer is as follows:

(15) $$\begin{array}{c} \begin{array}{c} H_{0}=x_{u},\\ H_{i}=\delta \left(H_{i-1}W_{i}+b_{i}\right),i=1,2,...,M-1,\\ q_{u}=\delta \left(H_{M-1}W_{M}+b_{M}\right), \end{array}\\ \begin{array}{c} H_{0}=x_{v},\\ H_{i}=\delta \left(H_{i-1}W_{i}+b_{i}\right),i=1,2,...,M-1,\\ p_{v}=\delta \left(H_{M-1}W_{M}+b_{M}\right). \end{array} \end{array}$$

The inner product is used to predict the missing score, and the end-user score characteristics and item score characteristics are trained through the following loss function, such as the following formula:

(16) $$\begin{array}{c} \min_{W_{u},W_{v},w}\frac{1}{2}\underset{R_{i,j}\in R}{\sum }\left(R_{i,j}\right.-\delta {\left(R_{i},R_{j}|\left.W\right)\right)}^{2}+\lambda \left\|W\right\|\text{.} \end{array}$$

Attention mechanism is a method to simulate human visual behavior pattern. In the field of natural language processing, attention mechanism can represent the weight of each text in the input text sequence. Attention mechanism only considers the internal influence of the text sequence and does not consider the external features. Self-attention mechanism is used to distinguish the contribution of each comment to the overall evaluation characteristics of the project. The intrinsic vector of self-attention is expressed by the following formula:

(17) $$\begin{array}{c} A=softmax\left(W_{a}\right)\tanh\;\left(W_{b}O^{T}\right)\text{,}\\ O=\left(o_{1i},o_{2i},...,o_{di}\right)\text{.} \end{array}$$

The formula is as follows:

(18) $$\begin{array}{c} doci=AO_{0}\text{.} \end{array}$$

Then, it is sent to the full connection layer to get the final expression of the features of the item comment set.

(19) $$\begin{array}{c} DeepDoci=W_{0}\times doci+b_{0}\text{.} \end{array}$$

After LSTM training, the output user's new preference feature at time *t* is u-long short-term memory network training process as follows:

(20) $$\begin{array}{c} \left[f_{t},i_{t},o_{t}\right]=\sigma \left[W\left(u_{i\left(t-1\right)},H_{jt}*\;\log r_{jt}\right)+b\right]\text{,}\\ {\tilde{c}}_{t}=\tanh\left[W_{c}\left(u_{i\left(t-1\right)},H_{jt}*\;\log r_{jt}\right)+b_{c}\right]\text{,}\\ c_{*}=f_{t}^{*}c_{t}+i_{t}^{*}{\tilde{c}}_{t,}\\ u_{it}=o_{t}^{*}\tanh\left(c_{t}\right)\text{.} \end{array}$$

Initial feature combination: in the research of recommendation system based on the multi-source information fusion, the most direct and common method is to overlay the corresponding dimensions of comment feature and score feature vector. In this paper, this method is used as a combination of initial features.

(21) $$\begin{array}{c} U_{initial}=p_{u}+DeepDoc\text{,}\\ I_{initial}=q_{v}+DeepDoc\text{.} \end{array}$$

High order feature combination: as mentioned in the previous paper, the high-order feature fusion of the model in this paper draws on the feature cross term in the factorization machine, which can simplify the calculation, such as the following formula:

(22) $$\begin{array}{c} \overset{n}{\underset{i}{\sum }}\overset{n}{\underset{j=i+1}{\sum }}<v_{i},v_{j}>x_{i}x_{j}=\frac{1}{2}\overset{n}{\underset{j=i+1}{\sum }}\left(\left(\overset{n}{\underset{j=i+1}{\sum }}v_{if}x_{i}\right)-\overset{n}{\underset{i}{\sum }}v_{if}^{2}x_{i}^{2}\right)\text{.} \end{array}$$

In this paper, user characteristic data and project feature data from different sources are spliced into two long vectors, such as the following formula:

(23) $$\begin{array}{c} L_{u}=p_{u}⊗DeepDoc\text{,}\\ L_{i}=q_{v}⊗DeepDoc\text{.} \end{array}$$

The user's different source feature stitching vector and the project's different source feature stitching vector are calculated by imitating the cross-feature term of the factor decomposition machine, and the high-order fusion features of the user and the project are obtained.

(24) $$\begin{array}{c} U_{high}=\frac{1}{2}\overset{n}{\underset{j=i+1}{\sum }}\left({\left(\overset{n}{\underset{j=i+1}{\sum }}v_{if}x_{i}\right)}^{2}-\overset{n}{\underset{i}{\sum }}v_{if}^{2}x_{i}^{2}\right),x_{i}\in L_{u}\text{,}\\ I_{high}=\frac{1}{2}\overset{n}{\underset{j=i+1}{\sum }}\left({\left(\overset{n}{\underset{j=i+1}{\sum }}v_{if}x_{i}\right)}^{2}-\overset{n}{\underset{i}{\sum }}v_{if}^{2}x_{i}^{2}\right),x_{i}\in L_{u}\text{.} \end{array}$$

After getting the initial and higher-order features, the final feature vector of the model is obtained by splicing strategy.

(25) $$\begin{array}{c} DeepU=U_{initial}⊕U_{high},DeepU\in R^{k+k\prime }\text{,}\\ DeepI=I_{initial}⊕I_{high},DeepI\in R^{k+k\prime }\text{.} \end{array}$$

In order to verify that the proposed model has higher recommendation performance advantages, this paper selects a real recommendation system data set with both rating data and comment data. Due to the fusion of multi-source features, the sparsity of rating comments and the length and quality of comment texts jointly affect the final prediction accuracy. Based on this, this paper selects data sets with different data orders and comment text quality as control experiments. The data sets of the actual replication system selected in this paper are as follows, and the data size and annotation text quality are introduced, respectively. In order to verify the validity of the white paper model, the public data set Amazon 5-core reviewed by Amazon uses toys and games, kindle store, and digital music. These are called as TG, KS, and DM. There are various sizes of these topics and datasets. The data set contains user IDs and project IDS, evaluation value, and comment value. The data set information is shown in Table 2.

It can be seen from Table 2 that although the number of users and the total number of items in the three real data sets selected in this experiment are very large, they belong to the category of large data sets, but they are very sparse. In the traditional matrix decomposition method, the matrix filling method is usually used for the sparse matrix, and the score and comment data feature fusion method is used in this model to overcome the high data sparsity problems caused.

It can be seen from Table 3 that although the data sparsity of the DM dataset is the highest, the length and quality of text reviews of the DM dataset are the highest in the three datasets. In the following experimental analysis, this paper will try to analyze the data sets with high text quality and improve the recommendation accuracy in the recommendation model with a more accurate text feature extraction module. The comparison of the proposed model with other benchmark models on three real datasets is shown in Table 4.

In order to prevent the accidental results of the experiment, this paper selects HR, NDCG, and other evaluation indicators suitable for recommended list category and carries out the test under the KS dataset. The results are shown in Table 5.

The experimental results show that this model has the same performance advantage in HR, NDCG, and other evaluation indicators compared with other models. Therefore, the multi-source feature hybrid recommendation model based on deep learning proposed in this paper has achieved ideal results in prediction accuracy and recommendation accuracy.

## 4. User-Centered Interaction Design

### 4.1. User-Centered Design

User-centered design needs to consider users at all stages of product development. The requirement of user software interface is an independent and important research field. By completing the design of software interface, the interface design of “user-centered” design concept is realized, and the interaction between human and computer can be realized, which can improve the acceptability. Improve the process and comfort and improve user satisfaction and subjective experience and improve the market competitiveness of products. The user-centered design concept no longer follows the development of product functions, but first discusses the logic of interface development and then allows users to learn, train, and adapt to the product in the form of interaction and logical working mode. However, based on the needs and experience of users, product plans are made, design and development work are the center, user requirements and experience are the center links to consider user actions, perceptions, psychology, experience, etc.

User-centered product design is very helpful to the design of “future products” and to the overall planning of a series of products. User centered design is not the beautification design of external packaging and interface but a revolutionary design, which realizes the whole process of user using the product through cognition, learning, use, psychology, and emotion. In the era of cloud services and big data, users can pay more attention to their task objectives, so that users can focus on learning costs. The purpose of users of the product is not to “learn” troublesome but to complete tasks efficiently and comfortably. Product function identification, learning, and understanding must be simple and easy to learn. It is the best to understand “what” and “use method” at a glance, take an example to introduce the Internet. If new users do not know the details of different products, the simplicity and ease of learning is an important factor affecting the choice of specific products.

User survey is not only helpful to product design, development, application, and sales but also helpful to product users. For the company, the user survey in the initial stage can provide more clear objectives and boundaries of the scheme design, reduce the repetition of design, save the development cost and resources in the later stage and greatly improve the possibility of obtaining users' trust and preference after the products are produced. Development is complete. For users, the product design can be closer to the actual needs through complete investigation and analysis. For users, this product is easier to master, easy to use, high efficiency, and to solve practical problems. If there is no clear and detailed knowledge, background, questions, user preferences, habits, needs and related business, and business objectives, even if creative designers, imagination, and product design are difficult to meet the user's needs, the user will be more difficult to use and less comfortable in the process of using, which will not be recognized by users and affect the promotion and use of products.

Even if users are provided with detailed specifications of mobile phones, tablet computers, navigators, tools, and other products, even if they “do not use” manual products, users who are used to “self-help” products in the actual use process can also learn how to use the website through operation forms and bricks, and no users have learned how to use the website through participating in the training and seminar products method. In the process of learning and using, users often have to experience a lot of difficulties and pain. However, because the user cannot skillfully use, easy to use, and other reasons, rarely complain to the product designer. Users may directly convert to learning and using simple competitive products, and they can enter a browser that completely conforms to the facts and have a friendly attitude towards this browser and use it for a long time.

**Figure 2:**
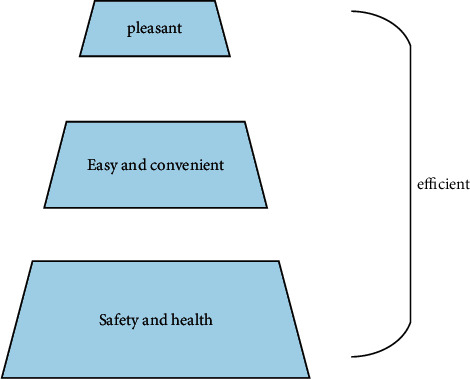
User experience design principle and its relationship.

According to the relevant research materials of ergonomics, it can be learned that this subject is mainly used to improve the people's living environment and work efficiency, so as to make life and work find the optimal state. Therefore, the core value of interaction design is to ensure the safety of information, the convenience of information transmission, the comfort of life, and the efficiency of work. User experience design principle and its relationship is shown in Figure 2.

### 4.2. User Research Methods

Through the description of the user survey method, it can provide the guidance of feasibility in the specific process of multi-level and multi-angle interactive standby design and the introduction, summary, and evaluation of the design scheme. In the specific implementation of user extension design, the survey methods commonly used include focus group, contextual interview, usability study, and user testing in the development process. The purpose of the investigation is to ensure the following: (1) The correct quotation of user experience at the beginning of design. (2) Understand the real expectations and goals of users. (3) If the functional core can be changed at a low cost, the design will be changed. (4) Ensure the expansion of the user interface, so as to make the user use the process more convenient and faster.

### 4.3. Evaluation of User Experience

User-centered interaction design can be interpreted as a multi-stage process problem. In this process, designers not only need to investigate, analyze, and investigate users but also need to predict how users identify and use products. Improve the user resource management as the starting point of design optimization and then test and evaluate the design scheme through the user's test in the use scheme. Generally speaking, in the user experience test, qualitative and quantitative methods are mainly used to investigate the physiological, psychological, action, and other related indicators of users. Function can be used and operated effectively, easy to master, efficient operation, easy to use, and comfortable.

## 5. User Interaction Experience Art Design of the Mobile System Based on Situational Awareness

### 5.1. Design of the User Interaction Experience Model

**Figure 3:**
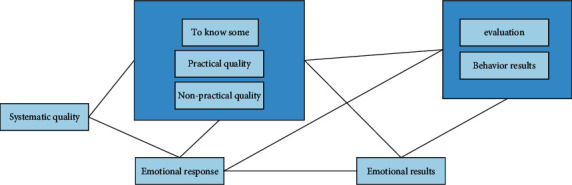
Basic user experience model.

In order to study the user extension of the whole interaction system, we must integrate all aspects and fully understand the user interaction extension. The basic user resource management process model (see Figure 3) will integrate relevant parts of user resource management to provide a basis for further investigation.

**Figure 4:**
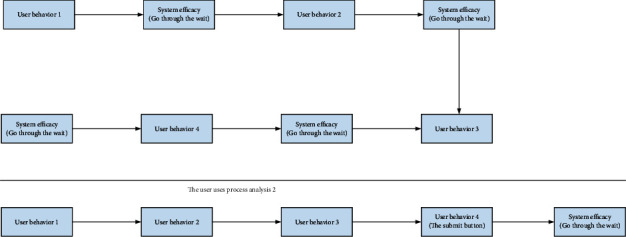
User use process analysis legend.

As shown in Figure 4, it shows that users will not waste the time waiting for system verification after each conversation and will not be interrupted due to the frequent system standby process, so they can focus on the conversation process with the actual business.

### 5.2. Analysis of Human-Computer Interaction

**Figure 5:**
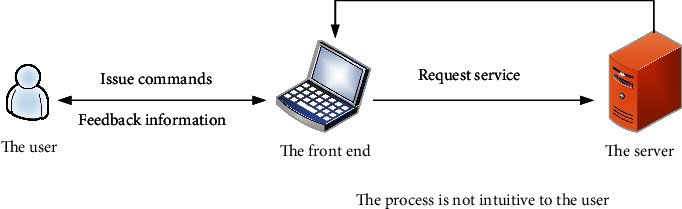
Interaction between the user and the system.

In daily life, the interaction principles of most websites, clients, and application products are as follows. The user executes the instruction through the interface. The front end of the product receives the user command and sends it to the server. The server processes the request and displays the data to the user interface. In this process, the communication interaction between the front-end interface and the server are not recognized by users, and the time spent in various network communication conditions is different, which has a certain impact on user experience. In addition to technical factors and objective environmental factors in the design process of the human-computer interaction system, we need to design the interaction process according to the user-centered design concept, so that users can feel that the whole product system is trying to have. His instructions must be followed and the task must be finished immediately. Through our design scheme, users are familiar with and understand the system, and users have basic psychological expectations for waiting time. Most importantly, users must provide a waiting experience and not something boring, relaxed, and happy. Interaction between user and system is shown in Figure 5.

Generally speaking, users expect the product to be easy to master, easy to use, and efficient. They hope to understand, master, and use these products in a short time and learn and use these powerful and advanced products in their hearts. The site also said that if visited for the first time, the data would not be displayed. This is because users generally do not want to waste time on anxieties and waiting. From the perspective of users, the Internet generates a lot of garbage every day. If the website cannot provide valuable business or content to users quickly and effectively, users can close the website directly and then quickly “convey” valuable content, so as to find other websites. The convenience of closing pop-up windows is a habitual response of many netizens. This may be due to the slow page reading for a few seconds, or the user feels that the business is unclear or incomprehensible and needs to make great efforts to understand “carefully.” It quickly determines the content of the site and how long it will be used. Designers must clearly show the functions of products according to users' cognitive habits within a very short period of time when users visit each page, so as to make it suitable for users' cognitive psychology, find useful information and judge the value. It can be accurately evaluated in a short time.

### 5.3. Human Computer Interaction Optimization Design Based on User Experience

**Figure 6:**
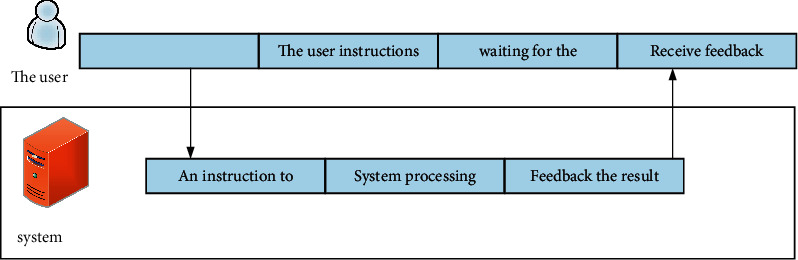
Example of front-end pre-operation reducing user waiting time.

By learning the communication between people, the computer builds action principles based on deep learning and applies them in human-computer interaction, so as to get the feedback of the system. The user sends instructions to the system, and the system receives and executes the instructions. The system processes the feedback information according to the user's expectations. The process of users waiting for feedback from the system is interactive waiting state. For users, the standby process is mainly used to wait for system processing and information communication. Boredom is an intuitive extension of user interaction standby, and it is also an important content of interactive standby design optimization. According to the user interface and operation rules, feedback can be found in the user interface and the operation process. Continuous execution of orders. As the name suggests, preprocessing of the front end is performed in advance to reduce the time required for the system to process the content later. For example, we are uploading some photos and videos. When users click the upload button, they can select the image or video content on the client to quickly compress and upload the action content. This is part of the upload completion operation. Click the upload button to continue to upload the next content. This can shorten the waiting time for users to upload. The interactive standby optimization scheme will maximize the use of client hardware performance ad optimize the interactive standby process. Example of front-end pre-operation reducing user waiting time is shown in Figure 6.

**Figure 7:**
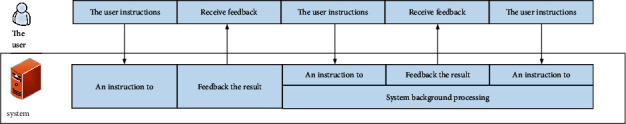
Example of interactive waiting through multitasking.

Multitask interactive standby design scheme is to use the performance function of hardware and software at the same time, that is multiple tasks. When the user clicks the link to upload, download, open the web page or video, the system will run in the background in the non-modal loading state, but the system also supports the user to continue the corresponding feedback operation. Example of interactive waiting through multitasking is shown in Figure 7.

## 6. Conclusion

In this paper, combined with the user's understanding, interaction habits, psychological emotions, analysis of the interaction process of interaction ability, through the design of the interactive business process, waiting for the expression and classification of necessary information, combined with user logic and psychology, in order to experience, design “intervention,” improve the dialogue process, improve the user resources of dialogue type standby, let the user “ignore” the dialogue type standby the “standby mode” is shortened.

## Figures and Tables

**Table 1:** Information gain value of each situation.

Contextual dimension	Information gain value
endEmo	0.17258
dominantEmo	0.13701
Interaction	0.02724
Mood	0.02485
Social	0.02059
Weather	0.01428
Time	0.00921
Season	0.00855
Physical	0.00839
Decision	0.00813
Daytype	0.00579
Location	0.00492

**Table 2:** Data set information statistics.

	Number of users	Number of projects	Ratings/comments	Sparsity (%)
TG	19412	11924	167597	92.75
KS	68223	61934	982619	97.67
DM	5541	3568	64706	99.67

**Table 3:** Statistics on the quality of data set comments.

	Average number of user comments	Average number of words commented by users	Average number of items commented	Average number of words in the commented text
TG	8.63	875.99	14.05	1426.09
KS	14.4	1616.22	15.86	1780.33
DM	11.67	2374.29	18.14	3687.21

**Table 4:** RMSE values of the experimental results of the comparison algorithm.

	svD++	HFT	DTMF	DeepCoNN	NARRE	OURS
TG	0.896	0.894	0.901	0.892	0.876	0.864
KS	0.784	0.789	0.787	0.784	0.781	0.778
DM	0.914	1.024	0.909	0.898	0.892	0.882

**Table 5:** The experimental results of NDCG and HR are compared.

	HR@5	HR@10	NDCG@s	NDCG@10
svD++	0.491	0.671	0.312	0.390
HFT	0.497	0.682	0.313	0.394
DTMF	0.492	0.669	0.312	0.391
DeppCoNN	0.548	0.712	0.384	0.436
NARRE	0.558	0.716	0.396	0.447
OURS	0.561	0.731	0.410	0.461

## Data Availability

The data used to support the findings of this study are available from the corresponding author upon request.
